# Response to ‘The factor structure of complex posttraumatic stress disorder in traumatized refugees’

**DOI:** 10.1080/20008198.2017.1307078

**Published:** 2017-03-31

**Authors:** Ad de Jongh, Iva A. E. Bicanic, Rafaele J. C. Huntjens, Henk A. L. Kiers, Mark van der Gaag, Arjen J. van Wijk

**Affiliations:** ^a^ Department of Social Dentistry and Behavioral Sciences ACTA, University of Amsterdam and VU University, The Netherlands; ^b^ School of Health Sciences, Salford University, Manchester, United Kingdom; ^c^ Institute of Health and Society, University of Worcester, United Kingdom; ^d^ National Psychotrauma Center Children and Youth, University Medical Center Utrecht, The Netherlands; ^e^ Department of Psychology, University of Groningen, The Netherlands; ^f^ Parnassia Psychiatric Institute, Den Haag, The Netherlands; ^g^ Department of Clinical Psychology, VU University Amsterdam and EMGO Institute for Health and Care Research, Amsterdam, The Netherlands

Dear editor,

With interest we read the recently published article ‘The factor structure of complex posttraumatic stress disorder in traumatized refugees’ by Nickerson and colleagues (2016). Studying the possible applicability of the complex posttraumatic stress disorder (CPTSD) construct on refugees is a matter of importance that might affect mental health approaches for this vulnerable population. Yet, the conclusions drawn by Nickerson et al. raise a number of questions and concerns that we would like to share with the readers of this journal.

The primary aim of the study was to assess the validity of the construct of CPTSD with a factor analytical approach in a sample of 134 refugees and asylum seekers living in Switzerland. Following the proposal for ICD-11, the authors assessed trauma exposure and administered a questionnaire consisting of 12 items, of which six pertained to the core symptom clusters of PTSD extracted from the Posttraumatic Diagnostic Scale (PDS; Foa, 1996), and the other six to the three additional symptom clusters that are supposed to be the core of CPTSD construct within ICD-11.

Regarding the validity of the measures, most CPTSD items were taken from questionnaires that are not trauma-related, which means that these items capture symptoms that may be related to other kinds of stressors. A possible alternative cause of distress within the group of refugees includes the consequences of conditions of displacement. As the authors state: ‘Refugees are displaced to unfamiliar environments, and may be unable to access important sources of support or established strategies for managing distress (e.g. work, leisure activities). These experiences may have an especially strong impact on the CPTSD domains of affect regulation, interpersonal relations and self-concept’. Obviously, many people who are living under the distressing circumstances of displacement may run a risk of feeling disconnected, developing low self-esteem and difficulties controlling their emotions. That is not what the CPTSD concept, which is explicitly trauma-related, is meant to convey. In addition, symptom endorsement may also result from comorbidity. For example, the items for disturbances in self-concept originate from the Hopkins Symptom Checklist (Mollica et al., 1992), and intend to measure depression. Comorbidity of PTSD and depression in refugees proves to be high (44–71%; Fazel, Wheeler, & Danesh, 2005) so refugees are likely to endorse these items. Nickerson et al. state that: ‘While many individuals with CPTSD reported these symptoms, so did many without CPTSD’. In other words: do these items really provide a valid measure of CPTSD in this sample?

Another important fact that casts doubts to the value of the results is that no clinical interviews were used, meaning that the participants of the study were not formally assessed for the presence of PTSD and CPTSD. For example, it could not be determined whether the reported traumatic events met the A-criterion (e.g. ‘imprisonment’, ‘forced separation from family members’, ‘lack of shelter’ and ‘brainwashing’). Accordingly, only a ‘probable diagnosis of PTSD’ was established. The authors reported that this probable diagnosis referred to less than 20% of the participants. A probable error in reporting is confusing in this context as the *N = *70 probable PTSD ‘diagnoses’ seems to include PTSD as well as CPTSD cases.

The improper assessment procedure may also have had negative implications for estimations concerning the prevalence of CPTSD in the target group. The authors reported the ‘probable’ prevalence of CPTSD among the refugees on the basis of questionnaires consisting of items that were taken from various questionnaires. However, claiming a diagnostic status based upon such method is premature. Related to this, the authors mention a series of studies (De Jong, Komproe, Spinazzola, Van der Kolk, & Van Ommeren, 2005; Morina & Ford, 2008; Palic & Elklit, 2014) to substantiate their argument that CPTSD may be particularly relevant to refugee groups. Unfortunately, they failed to mention that, in the cited studies, CPTSD prevalence was found to be low when assessed by using diagnostic interviews.

Maybe the most important point of critique relates to the conclusions of the authors that the findings indicated that the two-factor higher-order solution evidenced the best model fit, which they considered as support for the conceptualization of PTSD and CPTSD being separate constructs. As a measure of the degree of distinctiveness of the two factors PTSD and disturbances in self-organization (DSO) in the two-factor model, the authors report: ‘In the two-factor model, the correlation between PTSD and CPTSD was 0.84 (*p* < .001)’. An exceptionally strong association usually indicates that the factors virtually measure the same construct. Additionally, there seems to be another reporting or statistical error here, as CPTSD is not a factor in this model. The fact that the Chi^2^-value of the one-factor model was significant, and not significant for the two-factor model, cannot be considered as evidence that the second model fits better than the other. That is, if the idea was to test the significance of the difference in model fit, then, for instance, a Chi^2^ difference test should have been carried out. The authors, however, do seem to realize the limitations of Chi^2^ testing procedures, and additionally reported a series of other fit measures for both models. These all indicate a better fit for their particular two-factor model than for the one-factor model. However, the differences are rather small and offer a slim basis to conclude that their particular two-factor model tested should be preferred above the one-factor model. This is all so because the sample size is small, and other samples could easily give quite different outcomes due to sampling fluctuations alone. Furthermore, it should be noted that, even if the sample would be sufficiently large to increase the chance of ruling out the effects of sampling fluctuations, the chosen model fitting strategy has a fundamental flaw because there is an infinite number of models that could be tested on these data, some of which might even do better. Testing only two models can, in the best case, only lead to a clear preference for one of these, but this can never be used to claim that the best of these models is a good representation of the underlying state of affairs. In other words, the current study by no means shows that the CPTSD construct is the indicated way to improve the diagnosis of trauma victims.

In conclusion, we detected a number of flaws that we consider as potential threats to both the internal and the external validity of the study results and, therefore, compromise confidence in the conclusions of the authors. More specifically, given the small sample size, and the way the probable diagnoses PTSD and CPTSD were established, as well as the small differences between the two models, the findings of Nickerson et al. do not lend much credence to the existence of two clear and distinguishable symptom profiles, PTSD and CPTSD, nor do they provide support for the inclusion of a separate diagnosis for CPTSD in the ICD-11. Even if we were to take the results as indicating unequivocal evidence for the CPTSD construct (i.e. a two-factor model of PTSD and DSO), this would not imply different patient groups or different treatment implications for those with PTSD and CPTSD. To this end, the data certainly do not justify the utility of stabilisation interventions, such as Skills Training in Affective and Interpersonal Regulation for those suffering from symptoms of CPTSD. The deployment of such phase-based interventions for adults with CPTSD has recently been disputed (De Jongh et al., 2016), based upon the fact that well-designed studies directly comparing trauma-focused treatments, with and without a preceding stabilization phase, are lacking, and that the evidence from a number of recent studies show that trauma-focused therapies can be effective in a wide range of PTSD patients, including those with complex presentations (De Jongh et al., 2016; Ehring, Morina, Wicherts, Freitag, & Emmelkamp, 2014). Therefore, statements made by Nickerson et al., like ‘The finding that CPTSD is prevalent in traumatized refugees may point to the development and implementation of specific psychological interventions’, are premature and not consistent with the current scientific literature on this matter.
